# Investigation on spatial variability and influencing factors of drinking water iodine in Xinjiang, China

**DOI:** 10.1371/journal.pone.0261015

**Published:** 2021-12-17

**Authors:** Zhen Yang, Chenchen Wang, Yanwu Nie, Yahong Sun, Maozai Tian, Yuhua Ma, Yuxia Zhang, Yimu Yuan, Liping Zhang

**Affiliations:** 1 State Key Laboratory of Pathogenesis, Prevention and Treatment of High Incidence Diseases in Central Asia, Xinjiang Medical University, Urumqi, China; 2 Center for Disease Control and Prevention of Xinjiang Uygur Autonomous Region, Urumqi, China; 3 College of Public Health, Xinjiang Medical University, Urumqi, China; 4 Center for Applied Statistics, School of Statistics, Renmin University of China, Beijing, China; 5 College of Medical Engineering and Technology, Xinjiang Medical University, Urumqi, China; 6 Department of Pathology, Karamay Central Hospital of Xinjiang Karamay, Karamay, Xinjiang Uygur Autonomous Region, China; 7 Department of Clinical Nutrition, Urumqi Maternal and Child Health Institute, Urumqi, China; 8 Department of General Practice Medicine, Xinjiang Corps Hospital, Urumqi, China; Soil and Water Resources Institute ELGO-DIMITRA, GREECE

## Abstract

**Background and objectives:**

Xinjiang is one of the areas in China with extremely severe iodine deficiency. The health of Xinjiang residents has been endangered for a long time. In order to provide reasonable suggestions for scientific iodine supplementation and improve the health and living standards of the people in Xinjiang, it is necessary to understand the spatial distribution of iodine content in drinking water and explore the influencing factors of spatial heterogeneity of water iodine content distribution.

**Methods:**

The data of iodine in drinking water arrived from the annual water iodine survey in Xinjiang in 2017. The distribution of iodine content in drinking water in Xinjiang is described from three perspectives: sampling points, districts/counties, and townships/streets. ArcGIS was used for spatial auto-correlation analysis, mapping the distribution of iodine content in drinking water and visualizing the distribution of Geographically Weighted Regression (GWR) model parameter. Kriging method is used to predict the iodine content in water at non-sampling points. GWR software was used to build GWR model in order to find the factors affecting the distribution of iodine content in drinking water.

**Results:**

There are 3293 sampling points in Xinjiang. The iodine content of drinking water ranges from 0 to 128 μg/L, the median is 4.15 μg/L. The iodine content in 78.6% of total sampling points are less than 10 μg/L, and only that in the 3.4% are more than 40 μg/L. Among 1054 towns’ water samples in Xinjiang, 88.9% of the samples’ water iodine content is less than 10 μg/L. Among the 94 studied areas, the median iodine content in drinking water in 87 areas was less than 10 μg/L, those values in 7 areas were between 10–40 μg/L, and the distribution of water iodine content in Xinjiang shows clustered. The GWR model established had found that the effects of soil type and precipitation on the distribution of iodine content in drinking water were statistically significant.

**Conclusions:**

The iodine content of drinking water in Xinjiang is generally low, but there are also some areas which their drinking water has high iodine content. Soil type and precipitation are the factors affecting the distribution of drinking water iodine content, and are statistically significant (P<0.05).

## 1 Introduction

**Fig 1:**
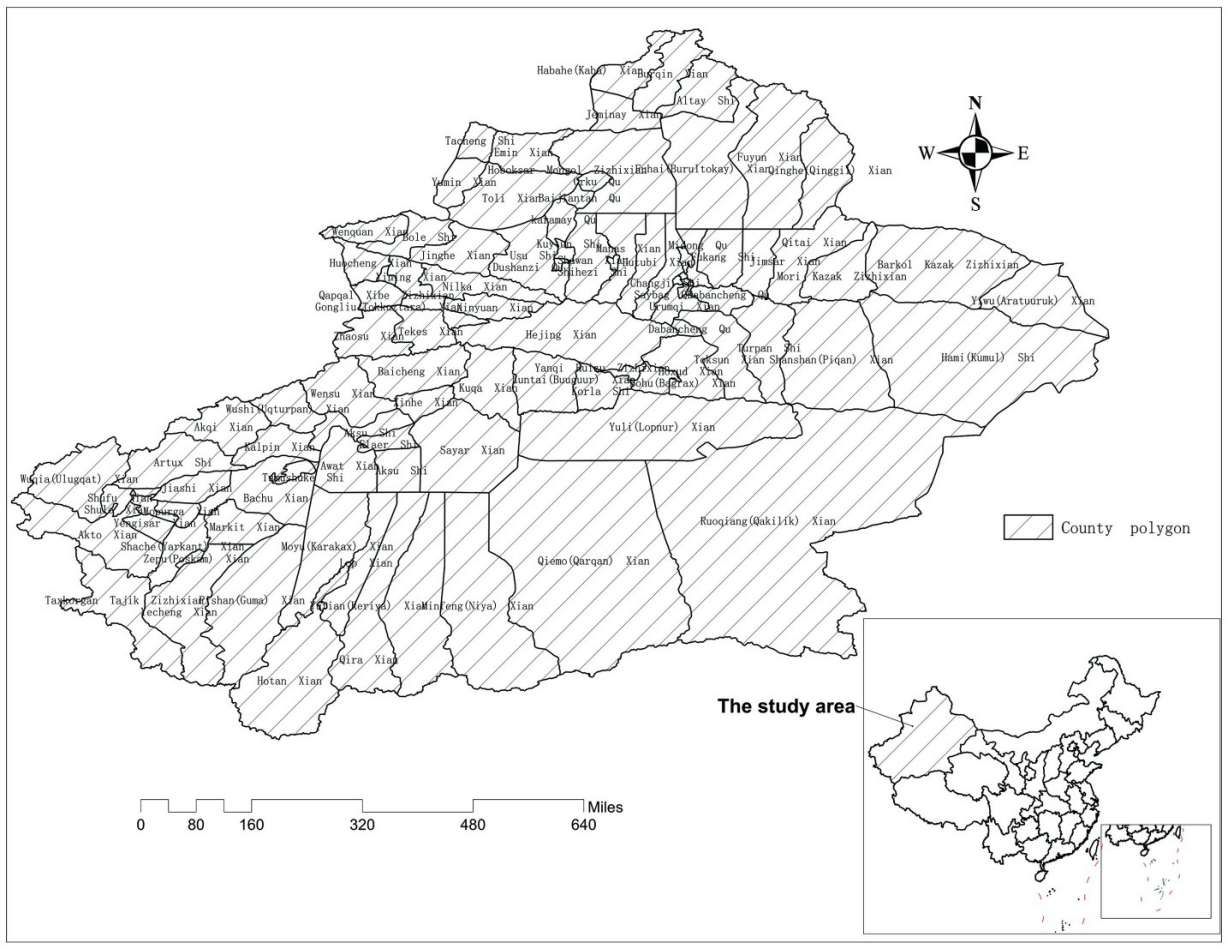
Study area: Xinjiang, China. The red area in the lower right corner represents the position of Xinjiang in China.

Iodine is an essential trace element for the human body, and its function is mainly to regulate physical and intellectual development and control energy metabolism through the synthesis of thyroxine [1]. The fetuses and children are the most sensitive groups to iodine deficiency. Iodine deficiency during pregnancy can lead to stillbirth, premature birth and congenital malformations [2]. Iodine deficiency in children may cause growth retardation, mental retardation, strabismus amblyopia, goiter and other symptoms [3]. Long-term iodine deficiency can also result in irreversible physical and mental damage to adults. Iodine deficiency is usually characterized by thyromegaly. Moreover, it is worth noting that excessive iodine intake can lead to hyperthyroidism, resulting in thyromegaly [4]. Xinjiang Uygur Autonomous Region (abbreviated as Xinjiang, shown in Fig 1), is located in northwestern China, in the center of the Eurasian Continent, (75°E~95°E and 35°N~50°N), and was once one of the most severely iodine-deficient provinces in China. The unique geographical characteristics and its distance from the ocean have resulted in low iodine content in its soils and water systems. Over the years, the local government has increasingly concerned about the current state of iodine deficiency. The government continues to invest heavily in establishing an iodine-deficiency disease monitoring system and to promote iodized salt. In addition, iodine supplementation for key populations such as children and women has alleviated the current effects of iodine deficiency. The rate of goiter among children decreased from 43.29% in 1995 to 0.72% in 2019 and the median urinary iodine level in children and pregnant women and the consumption rate of qualified iodized salt have steadily increased [5]. However, there are regional value differences in the distribution of urinary iodine level, and pregnant women in some areas are at the risk of iodine deficiency [6]. Therefore, attention should be focused on the iodine nutrition status of pregnant women in areas where the concentration of water iodine is less than 5 μg/L.

A cross-sectional survey of 2253 urinary iodine samples among adults who had lived in Xinjiang for more than three years showed that 34.4% were urinary iodine deficient [7]. The prevalence of hypothyroidism was significantly higher in the iodine-deficient group than in the iodine sufficient group. In Xinjiang, iodine deficiency disorder (IDD) remains a crucial public health problem for children, pregnant women, and the general population. In order to better prevent IDD, many researchers have been exploring the factors influencing human iodine nutrition levels.

At present, it is widely accepted that iodine primarily comes from the diet [8]. Iodine intake in diet is also the central aspect which local governments has made effort on. As the study has progressed, some researchers have turned their attention to iodine levels in drinking water. Liu L et al. found that the urinary iodine level of pregnant women was related to iodine content in local water [9]. Iodine in drinking water is also an important source of human body iodine intake. In order to provide reasonable suggestions for the scientific prevention of IDD, it is imperative to explore the spatial distribution and influencing factors of iodine content in drinking water. Kangmei J et al. investigated the distribution of water iodine content in Gansu, China in 2017 [10], attempting to guide the prevention and control of locally endemic diseases.

The traditional linear regression methods, such as ordinary least squares (OLS) regression, obtain the global relationship between dependent variable and explanatory variables without considering the spatial factor. Whereas, the Geographically weighted regression (GWR) model takes spatial variations into account [11]. Moreover, the GWR model weights nearby samples more than distant samples and includes samples from defined neighborhoods in the calculation. GWR results depend on observations close to the subject point, revealing the relationship within the neighborhoods [12]. GWR has been widely used in geography, meteorology and economic territory [13–15]. However, with its unique advantages, GWR model is now being applied to the medical field, especially in public health. In 2016, Das S. et al. studied the factors affecting the incidence of AIDS in Columbia, USA. They put forward targeted AIDS prevention programs according to the characteristics of each district in the United States of America [16]. Sannigrahi S. et al. investigated the relationship between socio-demographic composition in different European regions and deaths due to COVID-19 [17]. They found the novel influence factor of COVID-19 death rate, and obtained the satisfactory result.

The structure of this paper is as follows. First, this study systematically describes the current iodine content in drinking water in Xinjiang, examines the iodine content distribution and aggregation characteristics. Additionally, GWR model was established to explore the factors that may affect the iodine content in water. Based on the research results, reasonable suggestions are provided for scientific iodine supplementation in Xinjiang.

## 2 Methods

### 2.1 Research area and data source

The study was conducted based on Xinjiang Centers for Disease Control and Prevention (Xinjiang CDC) data. In 2017, Xinjiang CDC conducted a survey of drinking water iodine in 94 districts and counties under the jurisdiction of 14 prefectures in Xinjiang (four districts were excluded because there were no independent CDCs). Every prefecture and city are divided into districts and counties as study areas. There are 94 study areas in total. The county and district CDC are responsible for collecting water samples. The iodine content of drinking water data in each study area was collected based on the median iodine content of drinking water in each township and sub-district (streets), and the water iodine content value of each town and sub-district refers to the median of sampling points within its jurisdiction. The method recommended by the National Iodine Deficiency Disorder Reference Laboratory of the Chinese Center for Disease Control and Prevention was adopted to detect water iodine in both iodine-deficient and high-iodine areas. The water iodine kit was purchased by Xinjiang CDC and produced by Wuhan Minting Biochemical Company.

### 2.2 Influence factors

Refer to relevant articles of factors affecting iodine content in drinking water [18], this study incorporates economic factors, including GDP per capita (yuan/person), geographical factors (soil type, soil organic matter content (%), altitude (meter), hydrogeological type, soil chemical properties), and meteorological factor (precipitation). There are seven factors in three aspects. The GDP per capita data are from the statistical yearbooks of various regions in Xinjiang. The soil type data is from China soil science database where the soil type is divided into 13 categories according to China Soil System Classification. Precipitation data is from Official website of China Meteorological Administration. Other relevant data are from National Basic Geographic Information Center of China. The detailed classification of influencing factors has been listed, and the value is assigned (Table 1).

**Table 1:** Detailed classification of some influencing factors.

Assignment value	Soil type	Hydrogeological type	Soil chemical properties
1	Takyr soil	Clastic rock pore and fissure water	Organic carbon type soil
2	Yellow soil	Low-water content Crystalline rock fissure water	Gypsum soil
3	Grey desert soil	High-water content loose rock pore water	Saline soil
4	Brown desert soil	Low-water content loose rock pore water	Carbonate soil
5	Chernozem	Middle-water content Crystalline rock fissure water	
6	Oasis Soil	Middle-water content loose rock pore water	
7	Calcareous soil		
8	Inland saline soil		
9	Swamp soil		
10	Chestnut soil		
11	Grey-brown Desert soil		
12	Gray meadow soil		
13	Brown calcium soil		

### 2.3 Geographically Weighted Regression (GWR) model

According to "Tobler’s First Law of Geography" proposed by Tobler [19]: Everything is spatially related, and the closer the distance, the greater the spatial correlation between things. Ordinary least squares (OLS) regression model cannot take spatial factors into account, for it will lose important information. Moreover, OLS regression model can only estimate the parameters globally and cannot show the characteristics of different regions. Therefore, GWR model is a better option for this study. GWR model is a spatial regression analysis method proposed by Fotheringham [20]. On the premise of considering the spatial dependence of variables, GWR model analyzes the influence of independent variables on dependent variables and estimates the parameters of each region.

### 2.4 Data analysis procedure

Excel software was used to establish database and input data. ArcGIS (version 10.8) software was used to perform the spatial auto-correlation analysis, mapping the distribution of water iodine content and visual model parameters. Global/Anselin Moran’s Index (Moran’s I) was used to determine the presence of spatial auto-correlation. GWR4 was used for geographically weighted regression analysis. Areas where the median iodine content in drinking water ranging between 5–10 μg/L are defined as iodine-deficient areas, while the content value severely iodine-deficient areas are less than 5 μg/L; low-iodine areas are between 10–40 μg/L; suitable-iodine areas are between 40–100 μg/L; high-iodine areas is greater than 100 μg/L.

## 3 Results

### 3.1 Spatial distribution of iodine content in drinking water

A total of 3293 water samples were tested in Xinjiang. The iodine content of drinking water ranges from 0 to 128.8 μg/L, the median value is 4.15. Among them, 58.31% were in severe iodine deficiency area, 20.29% in iodine deficiency area, 18% in low iodine area, and 3.4% in suitable iodine area and high iodine area. In 14 prefectures, Bortala, Turpan, Urumqi, Kizilsu Kirghiz, Hotan and Kashi sampling points had the highest proportion of iodine deficiency and severe iodine deficiency, accounted for 100%, 100%, 97.87%, 95.31%, 94.44% and 92.02%, respectively. Altay, Karamay, Bayingol, Tarbagatay, respectively 47.12%, 58.33%, 63.8%, 64.38% (Table 2).

**Table 2:** Number and proportion of sampling points with water iodine content less than 10 μg/L.

Prefectures	Total number of Sampling points	Number and percentage of 0–5 μg/L and 5–10 μg/L sampling points				
		0–5 μg/L	proportion (%)	5–10 μg/L	proportion (%)	Total proportion (%)
Bortala	19	6	31.58	13	68.42	100
Turpan	60	34	56.67	26	43.33	100
Urumqi	141	122	86.52	16	11.35	97.87
Kizilsu Kirghiz	192	157	81.77	26	13.54	95.31
Hotan	144	106	73.61	30	20.83	94.44
Kashgar	213	137	64.32	59	27.70	92.02
Ili	418	299	71.53	65	15.55	87.08
Changji	368	260	70.65	52	14.13	84.78
Hami	78	50	64.10	15	19.23	83.33
Aksu	608	348	57.24	131	21.55	78.79
Tarbagatay	640	265	41.41	147	22.97	64.38
Bayingol	163	69	42.33	35	21.47	63.80
Karamay	24	14	58.33	0	0	58.33
Altay	225	53	23.56	53	23.56	47.12
Total	3293	1920	58.31	668	20.29	78.60

**Fig 2:**
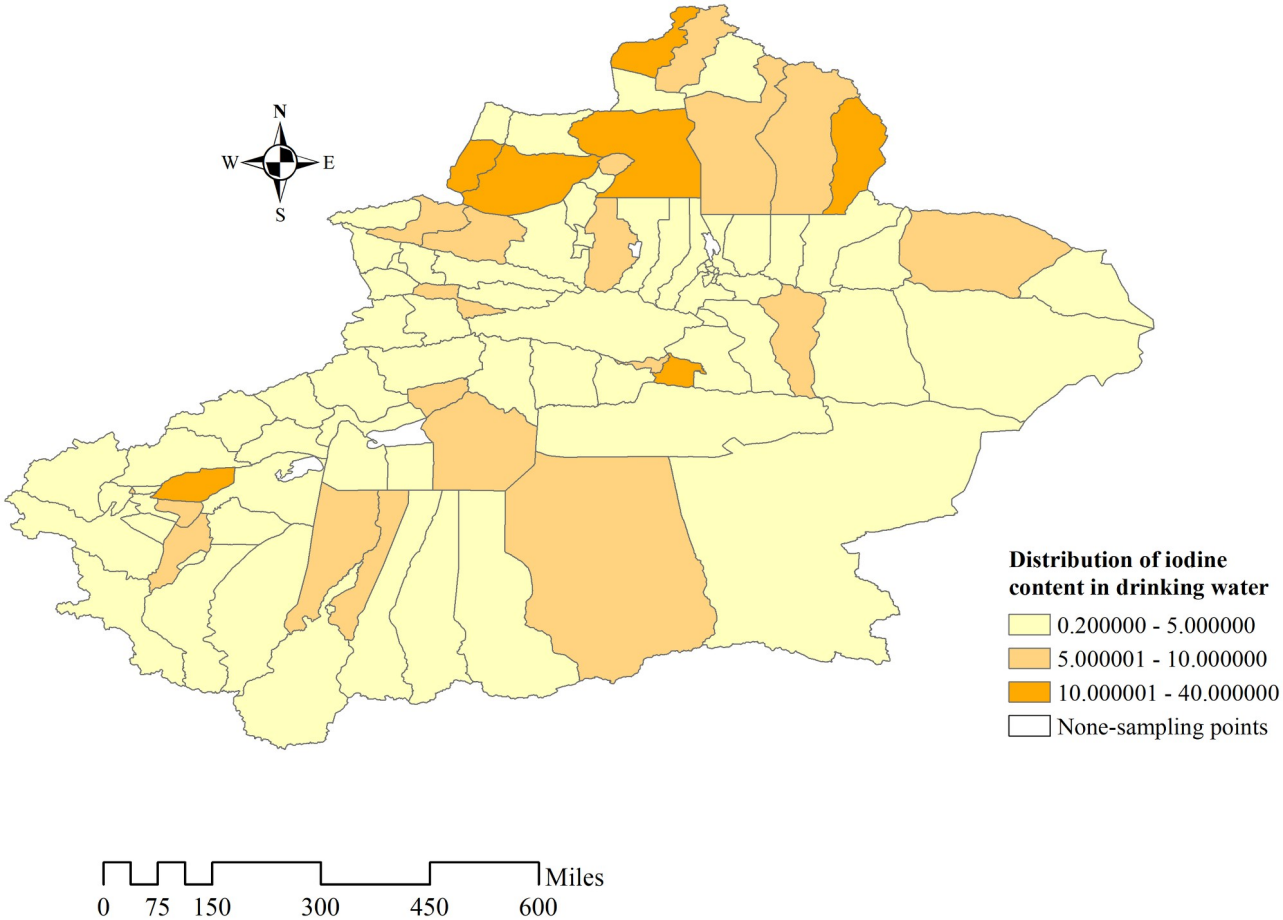
Distribution of iodine content in drinking water in Xinjiang. The median iodine content in water is displayed from light to dark color, and no sampling point is in the white area.

ArcGIS was used to describe the distribution of water iodine content in those 94 study areas (Fig 2). The iodine content of drinking water in Xinjiang is generally at a low level. There are 87 areas where the median iodine content is less than 10 μg/L (92.55%). There are 68 areas among low water iodine area where their iodine content is less than 5 μg/L (72.34% of total study area). There were only 7 areas with iodine content ranging between 10–40 μg/L (7.45%). There is no area that its iodine content exceeds 40 μg/L.

**Fig 3:**
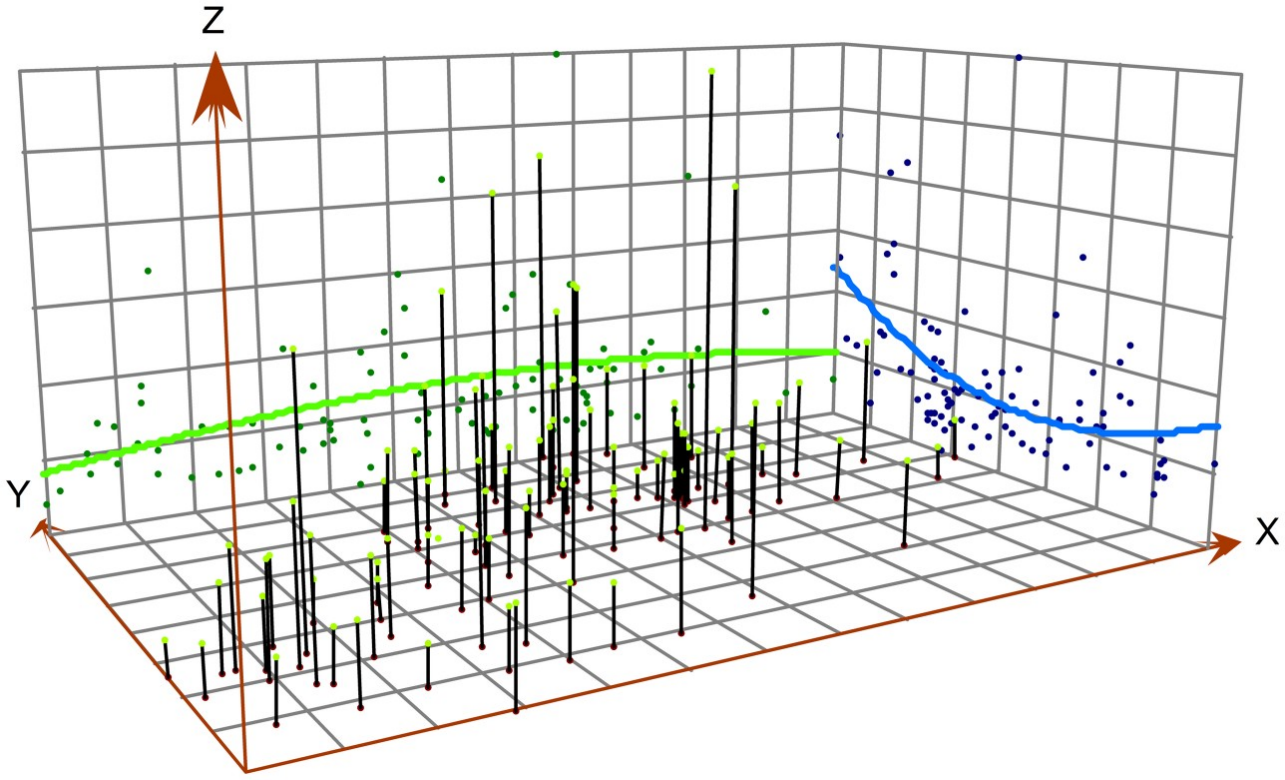
Trend chart. The Y-axis arrow points to the north, the X-axis arrow points to the east, and the Z-axis represents the iodine content of water. The blue line is the trend line in the north-south direction, and the green line is the trend line in the east-west direction. The black vertical line segment in the figure represents the water iodine content in each region. The longer the line segment, the higher the water iodine content.

The trend analysis chart was obtained after excluding the influence of local and random variation according to the principle of least square method, and thus can directly reflect the spatial change trend of iodine content in drinking water, facilitating the analysis of the change trend in the study area. The Y-axis arrow indicates that the direction is north, whereas the X-axis arrow points to east, the Z-axis represents the iodine content of water. According to the trend chart, the water iodine content in the north is the highest, followed by that in the south, and the lowest content value is in the middle. From east to west, the central part is slightly higher than the eastern and western parts. (Fig 3).

### 3.2 Spatial auto-correlation analysis

**Fig 4:**
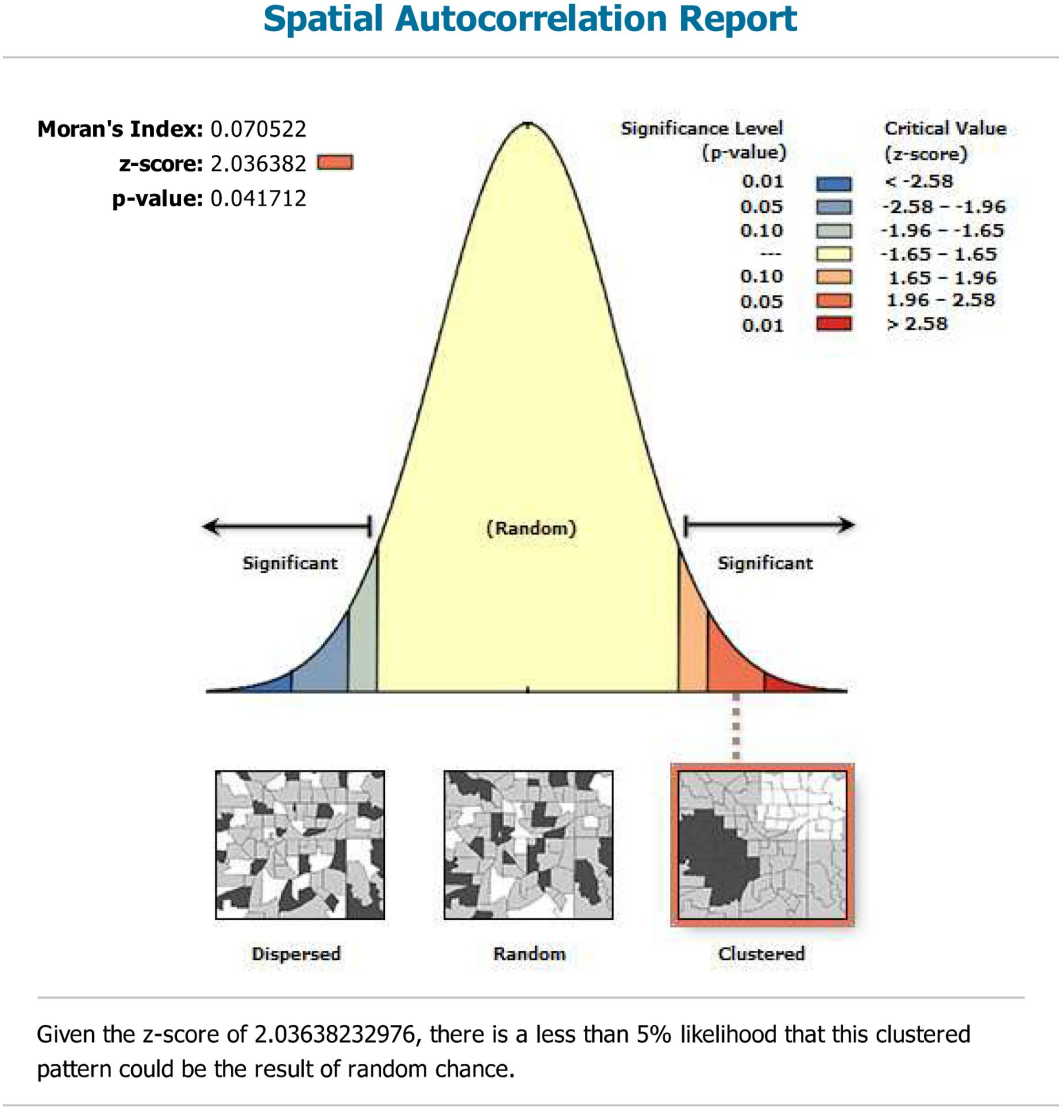
Spatial auto-correlation analysis report.

Spatial auto-correlation analysis is one of the spatial statistical analysis methods, mainly investigating whether there is a specific spatial dependence between data. Size and direction of spatial auto-correlation are measured by Global Moran’s Index. The value of Moran’s Index is generally between—1 and 1, and the value greater than 0 indicates that there is positive auto-correlation. The positive auto-correlation shows that high value is adjacent to high value, so is the low value; Moran’s Index less than 0 indicates negative auto-correlation, that is, high values are adjacent to low values. If Moran’s Index is close to 0, it will indicate that the spatial distribution is random and there is no spatial auto-correlation. Spatial auto-correlation analysis is conducted supported by ArcGIS. Spatial distribution of iodine content in drinking water in Xinjiang is not random (Global Moran’s I = 0.0705, p-value = 0.0417). It shows clustered distribution (Fig 4).

Global spatial auto-correlation explains whether there is an agglomeration in the research area, while local spatial auto-correlation explains its specific spatial location and form. Generally speaking, there are four forms of aggregation: High-High aggregation (H-H), Low-Low aggregation (L-L), High-Low (H-L) aggregation and Low-High (L-H) aggregation. In addition, other forms of distribution not showing aggregation, which are called random distribution, are not statistically significant. H-H aggregation means high local and surrounding values, while L-L aggregation implies low local and surrounding values. H-L aggregation means that the local value is high and the surrounding value is low while L-H aggregation means that the local value is low and the surrounding value is high.

**Fig 5:**
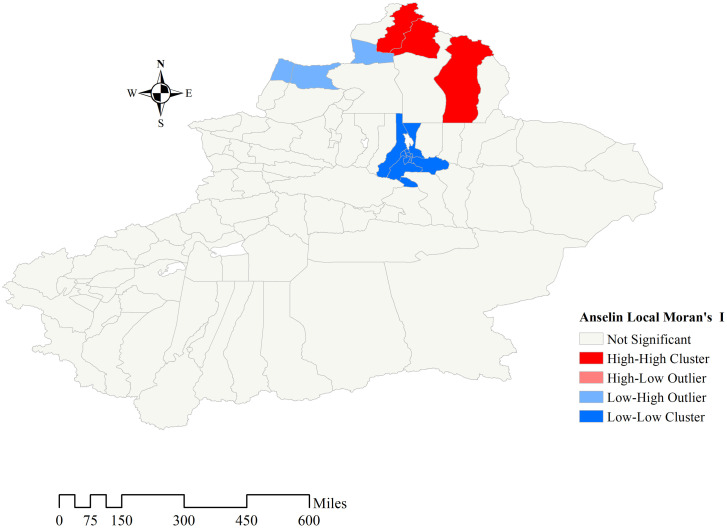
Anselin local Moran’s index distribution map. Different colors indicate that there are different aggregation patterns in this area, and light gray areas indicate that aggregation is not significant.

The specific local Moran index was calculated by Anselin Local Moran’s Index. The relationship between local and surrounding areas’ water iodine content was statistically significant in 15 of the 94 areas, plotted as a LISA clustering map (Fig 5). Fuyun County, Altay City and Buerjin County present H-H aggregation. L-L aggregation was discovered in Changji, Urumqi County, and seven districts of Urumqi. Tacheng City, Emin County, and Jeminay County exhibited L-H aggregation.

### 3.3 Discussion on spatial distribution based on Kriging interpolation method

The iodine content of drinking water in 1054 towns and streets in 14 prefectures of Xinjiang was recorded. 98 in Aksu, 57 in Altay, 92 in Bayingol, 20 in Bortala, 65 in Changji, 55 in Hami, 101 in Hotan, 167 in Kashgar, 12 in Karamay, 45 in Kizilsu Kirghiz, 94 in Tarbagatay, 33 in Turpan, and 92 in Urumqi, and 123 in Ili. It is verified that some townships are merged and rebuilt, and the actual township/street detection rate is 100%.

**Fig 6:**
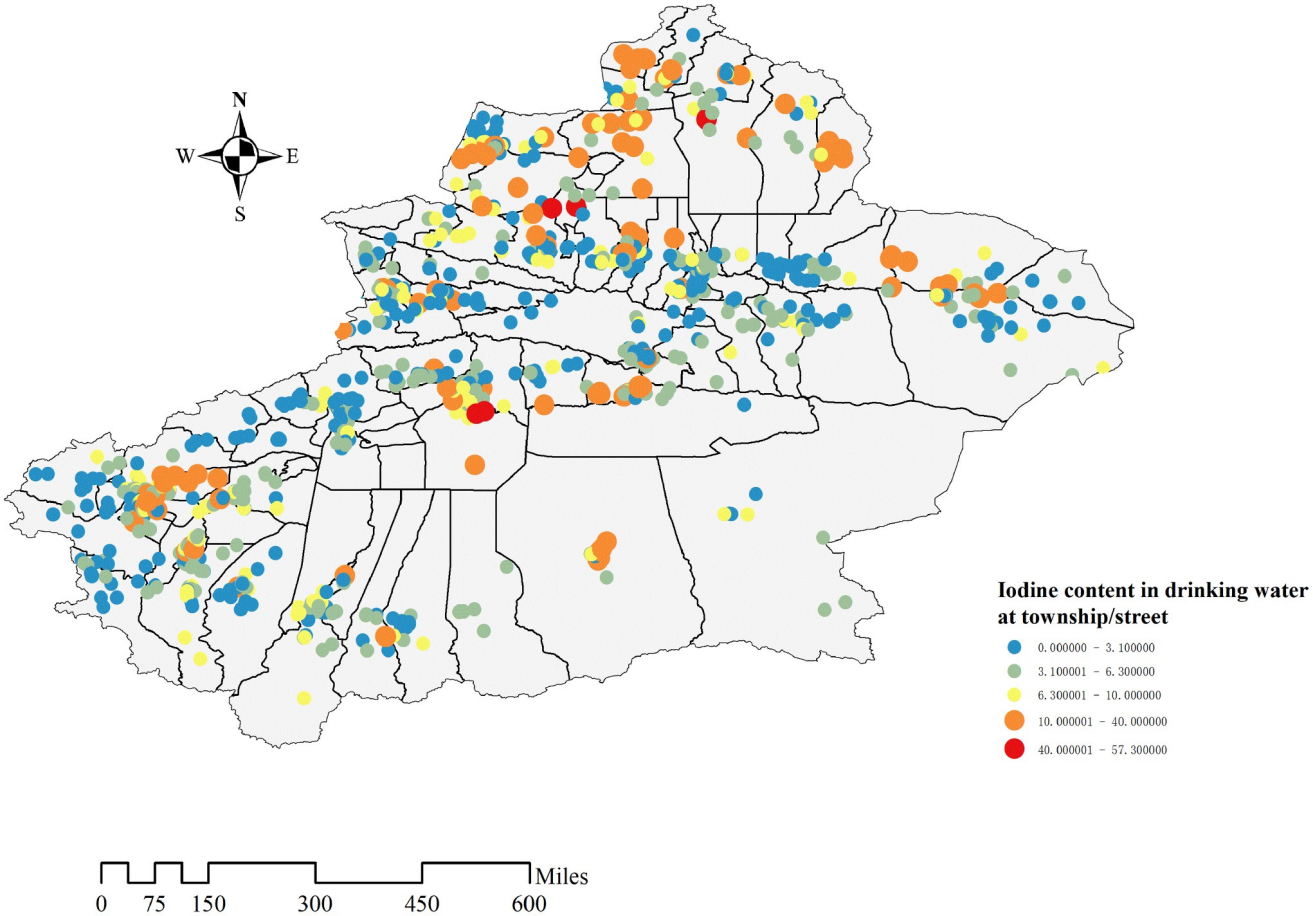
Iodine content in drinking water at township/street. The closer the color is to the red location, the higher the iodine content in the water, and there will be larger points to mark.

The spatial distribution of iodine content in drinking water from 1054 towns/streets in 14 study regions of Xinjiang was described precisely (Fig 6). The water iodine content of those towns/streets is expressed by the median water iodine content of sampling points under their jurisdiction, because the distribution of water iodine content is not normal distribution. Towns/streets with suitable water iodine content (40 μg/L-100 μg/L) and high-water iodine content (>100 μg/L) are marked with larger orange/red icons. 937 townships, accounting for 88.9% studied towns in Xinjiang has water iodine lower than 10 μg / L. 117 townships have content value higher than 10 μg/L, accounting for 11.1%, and there are mainly distributed in Tarbagatay, Altay, Bayingol and Kashgar, accounting for 21.37%, 17.95%, 16.24% and 13.68% of the total number of townships under their jurisdiction respectively.

**Fig 7:**
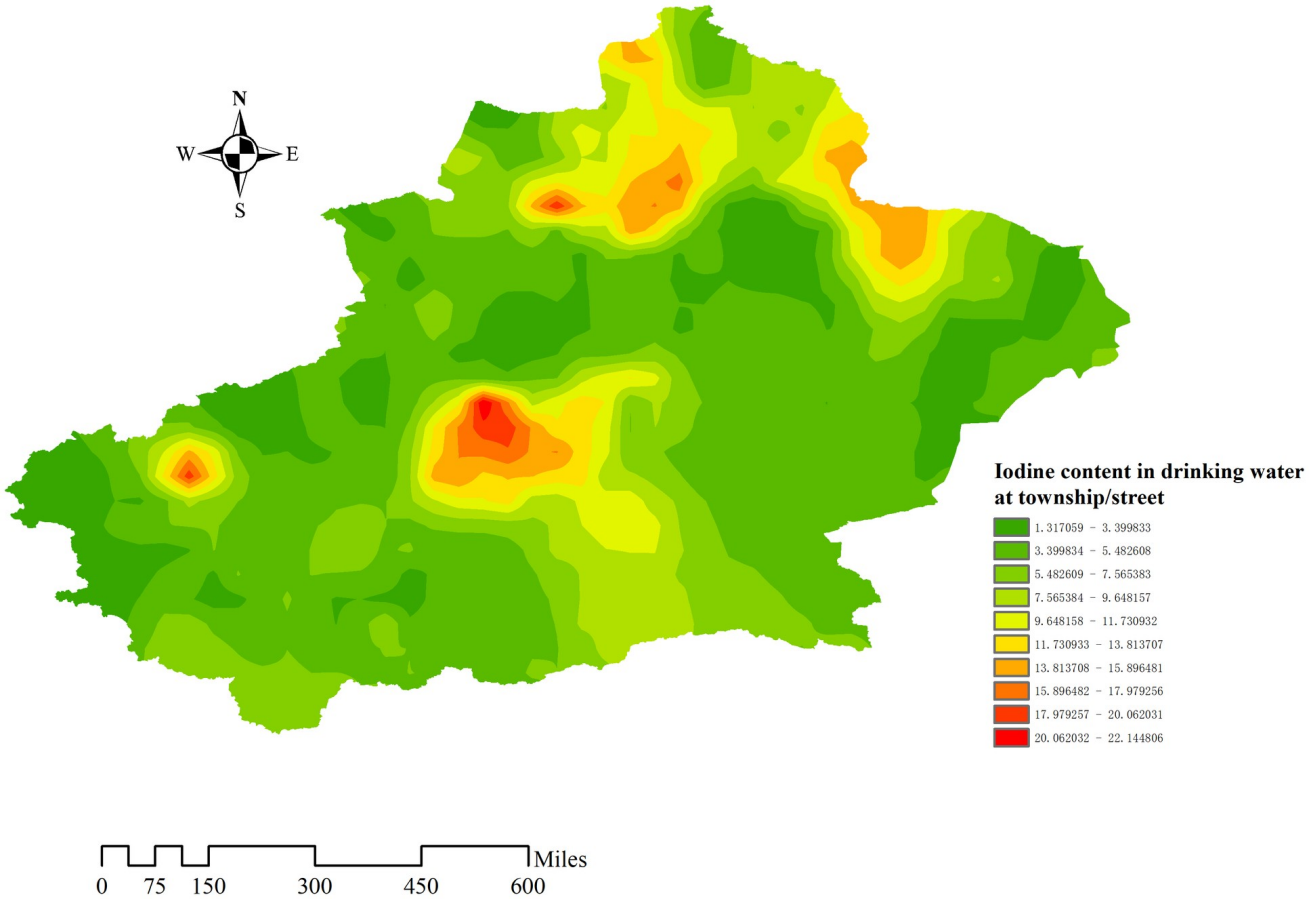
Kriging interpolation prediction map. Kriging interpolation method is used to predict the water iodine content in non-sampling areas. The closer the color is to dark red, the higher the water iodine content is, and the closer it is to dark green, indicating that the water iodine content is lower.

Kriging interpolation method is obviously more detailed than using a single number to represent the water iodine level of the whole area. However, we need kriging interpolation technique to predict the region not included in sampling points based on observed measurements. Before implementing the interpolation method, we need spatial auto-correlation analysis to judge whether there is spatial correlation in the iodine content of drinking water in towns and streets. The results show that the Moran’s Index is 0.2902 and the statistical test is significant (P = 0.0137). The value imply that the distribution of water iodine content shows aggregation. Kriging interpolation method is applicable to illustrate describe the distribution of iodine content in the region of non-sampling points (Fig 7), and is a spatial interpolation method based on spatial auto-correlation between regionalized variable theory and variogram [21]. Its essence is to utilize the original data of regionalized variable and the structural characteristics of variogram in order to make linear unbiased and optimal estimation of the regionalized variable at the non-sampling point. The results showed that the areas with higher water iodine content are mainly distributed in Bayingol in central Xinjiang and Karamay, Tarbagatay and Altay in northern Xinjiang.

### 3.4 Geographically weighted regression model

We selected GWR model to analyze the influencing factors of iodine distribution in drinking water. Science GWR model considers the spatial auto-correlation of variables (section 2.3). SPSS software is used to test the normality of water iodine content in various regions and carry out normal transformation, taking the iodine content of converted water as the dependent variable while taking economic factors, geographical factors and meteorological factor as independent variables, modeling and analysis. The model coefficients are calculated (Table 3) and draw the coefficient distribution map.

**Table 3:** Model coefficient.

variables	Min	Lower-Quartile	Median	Upper- Quartile	Max
Intercept	0.262574	0.343711	0.375865	0.604850	0.755750
Precipitation	-0.001241	-0.001014	-0.000934	-0.000715	-0.000468
Altitude	-0.073944	-0.040014	0.010530	0.022791	0.039172
GDP	-0.049091	-0.037370	-0.026634	-0.006992	0.003923
Soil type	0.012492	0.017911	0.031418	0.033609	0.039986
Soil organic matter content	-0.021111	-0.017911	-0.017407	-0.013845	-0.011252
Hydrogeological type	-0.025352	0.004289	0.016357	0.024013	0.041967
Soil chemical properties	0.026049	0.047288	0.058879	0.092603	0.103528

**Fig 8:**
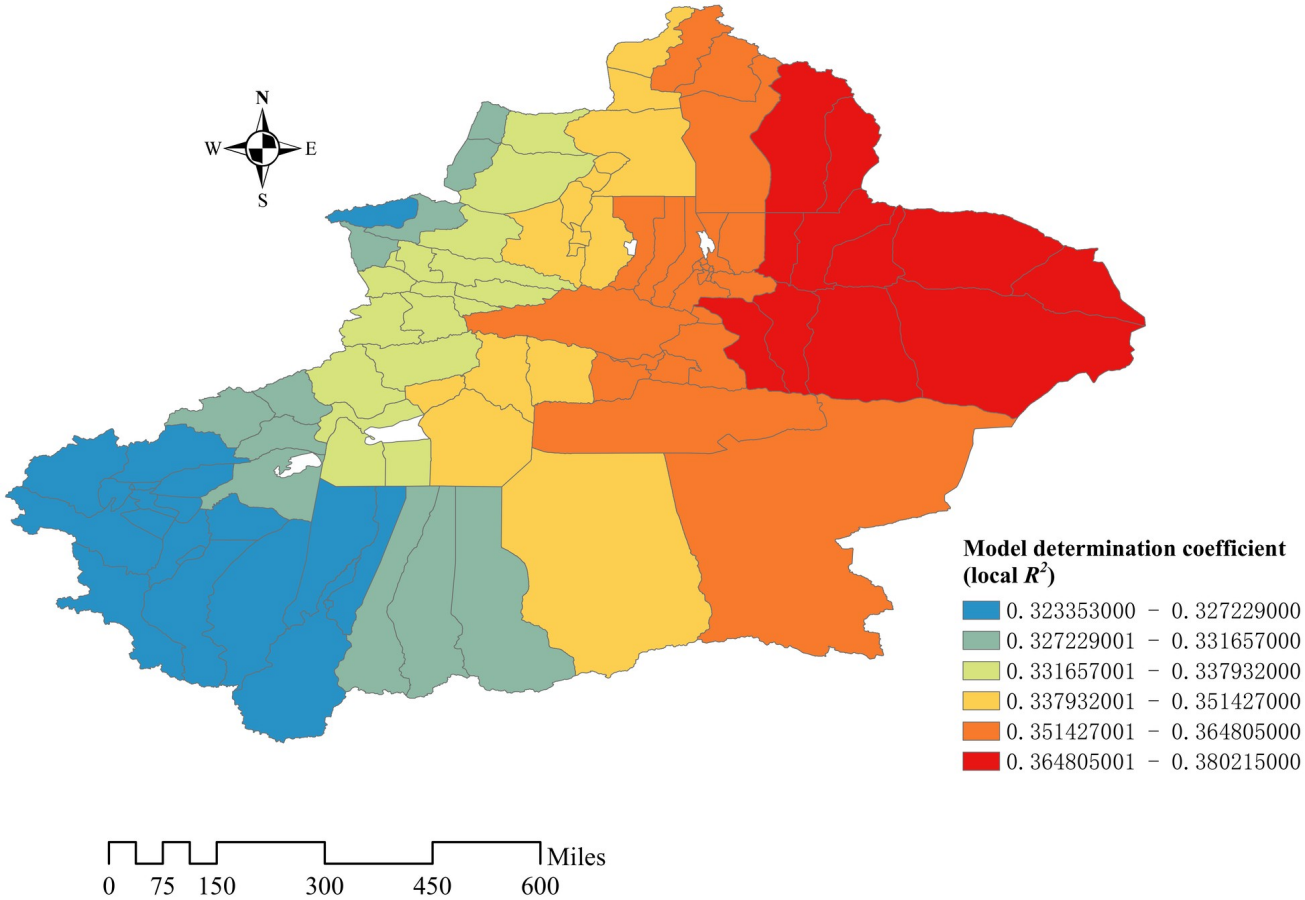
Coefficient of determination (*R*^2^) distribution. Blue, green, yellow, orange and red color represent *R*^2^ values from low to high.

Local *R*^*2*^ (coefficient of determination) was used to evaluate the goodness of fit of the model, the closer it is to 1, the better the fitting effect of the model. The value of *R*^*2*^ in this GWR Model is between 0.323–0.380. The spatial distribution of *R*^*2*^ shown on the map. The value increases from the west to the east of Xinjiang, this trend means that the model’s fitting effect is the best in the east of Xinjiang (Fig 8).

**Fig 9:**
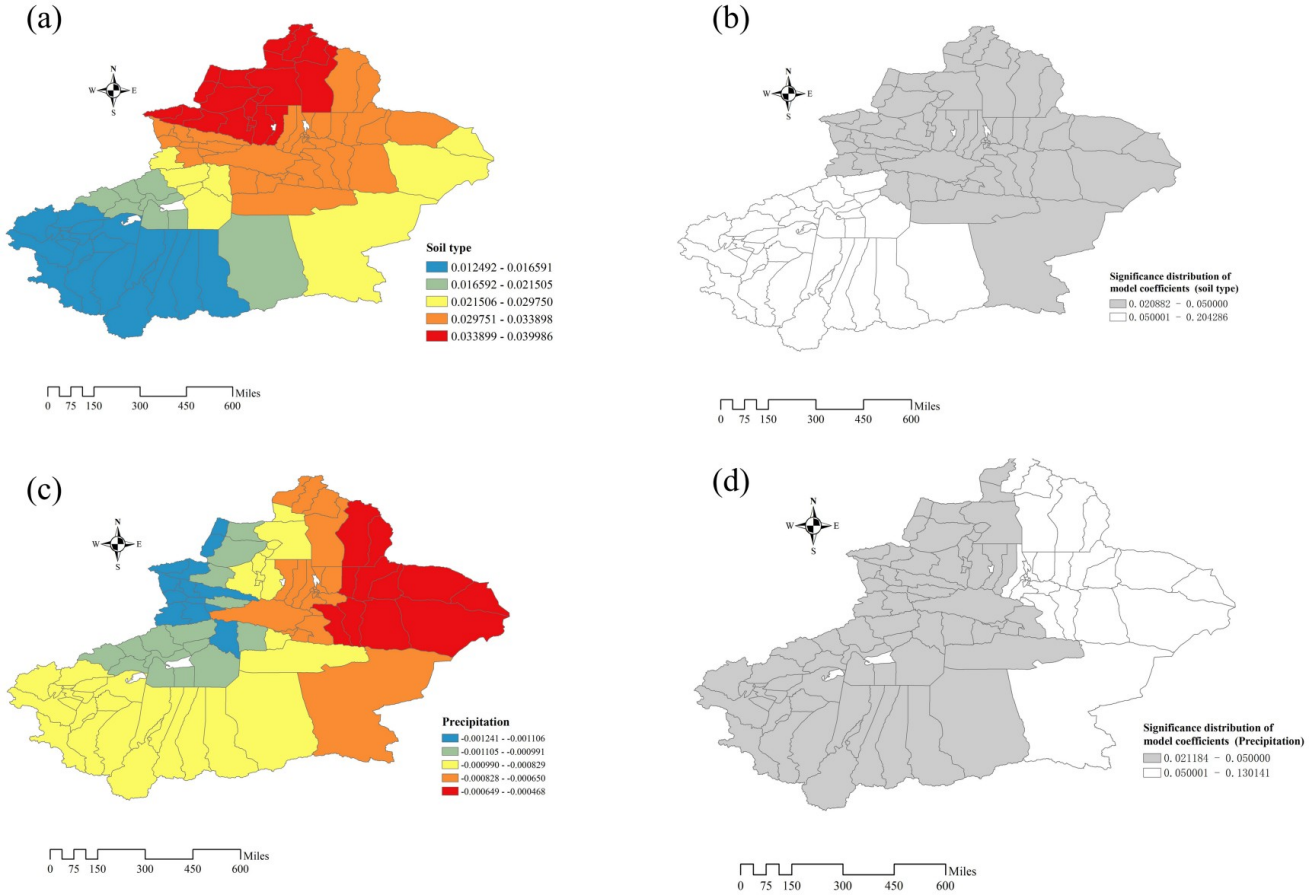
Model coefficient distribution and test statistics. In (a) and (c) different colors are used to represent different model coefficient levels, Blue is the lowest, and The closer the color is to red, the higher the coefficient. In (b) and (d), the model coefficient has statistical significance in the gray area.

GWR model fitting results show that, precipitation and soil type have statistical significance on iodine content in drinking water (P<0.05). Spatial distribution of soil types regression coefficients shows that the effect on water iodine content has evident spatial variability (Fig 9(a)). In the GWR Model, the coefficient of soil is greater than 0, which means that, with the increase of soil value, the content of water iodine increased gradually. Besides, the impact of soil type on water iodine content is enhanced in a gradient from south to north, which is statistically significant in Xinjiang’s eastern and northern regions (Fig 9(b)). The effect of precipitation on iodine content in drinking water also has obvious spatial variability, especially in the statistically significant central and western regions of Xinjiang, this influence gradually increased from west to middle (Fig 9(d)). It is worth noting that the coefficient here is negative, showing that with the increase of rainfall, the content of drinking water decreases (Fig 9(c)).

## 4 Discussion

Xinjiang is located in the middle of the continent, far away from the ocean. The water supply comes from glaciers, so the iodine content in the soil and water system is very low. This circumstance makes Xinjiang a distinct region in the world with severe iodine deficiency. Long term iodine deficiency may cause harm to the residents living here. Iodine deficiency disorder (IDD) is an endemic disease caused by iodine deficiency, this disease is widely spread and is a serious public health problem with regional characteristics in Xinjiang. IDD has caused serious constraints on poverty alleviation, economic development, national prosperity, population quality, and social development in Xinjiang. For a long time in the past, people living in Xinjiang have been suffering from iodine deficiency. Since 1995, the monitoring results have shown that after years of unremitting efforts, the prevention and treatment of iodine deficiency disease in Xinjiang has achieved great and positive effects. However, the risk of iodine deficiency disease still exists in some areas.

In this water iodine survey, about 78.6% of the sampling points had iodine content is lower than 10 μg/L in drinking water, while 3.4% of the sampling sites had iodine content higher than 40 μg/L. Against the background of general iodine deficiency, iodine content was adequate or even too high in a few areas, as shown in Figs 6 and 7. There may be different water iodine contents in the same area, and even in villages and towns belonging to the same county, the water iodine level may vary. Low or high iodine can cause damage to health. Excessive iodine intake may give rise to hyperthyroidism, hypothyroidism, goiter, and autoimmune thyroid diseases in some people [22]. At present, Xinjiang has greatly improved the current status of IDD through comprehensive iodine supplementation measures, but special guidance should also be given to areas with adequate and high iodine content level. Therefore, policies should be adopted according to the actual situation in different regions. For example, the use of iodized salt should be continuously promoted in iodine-deficient areas. Especially, Urumqi, which belongs to the L-L aggregation area of water iodine content. Bortala, Turpan, Urumqi, Kizilsu Kirghiz, Hotan, and Kashi have a high proportion of sampling points with water iodine content below 10 μg/L. More attention should be given to these areas with low water iodine content, and iodine supplementation should be increased for sensitive populations such as children and pregnant women living in these areas. The use of iodized salt should be reduced in areas with appropriate and high iodine content to avoid the harm caused by excessive iodine intake, areas including Altay and Bayingol. However, considering the complexity of water iodine distribution in Xinjiang, we speculate that the division of high-water iodine areas or low-water iodine areas is inaccurate. Therefore, the next step should be to take towns and cities as the smallest water iodine division unit, and divide them into low iodine zone, moderate iodine zone, and high iodine zone, and adopt different strategies based on local conditions according to the comprehensive and accurate distribution of water iodine. The shift from wide to precise iodine supplementation will bring the greatest benefits to the population in Xinjiang.

Xinjiang covers an area of 1.66 million square kilometers, with vast territory and complex topography. Each region has a unique natural environment and the iodine content in water is related to the geography. For example, in Fuyun, Altay and Buerjin, the drinking water iodine content presents the H-H aggregation. Ying Y. et al. concluded that these areas have water systems such as the Irtysh River, Ulungur River passing through, and the perennial scouring of the rivers is a fundamental reason for the high iodine content in drinking water [23]. In order to find out the factors affecting the distribution of water iodine, GWR model was established. It was found that both soil type and precipitation are statistically significant (P<0.05), and both of them might be factors affecting the distribution of iodine content in water. With the increase of precipitation, the iodine content in drinking water gradually decreases. This may be due to rainwater with very low iodine content diluting the iodine in the water, especially in Xinjiang, which is far from the sea, where the iodine content in rainwater is even less. Neal C. found that the iodine content in rainfall is lower than in cloud water, throughfall, stemflow, stream water, and groundwater [24]. The groundwater has the highest iodine content, and rainwater scouring causes iodine to sink deep into the soil. With the change of soil type, the content of water iodine also increases. The main soil types with low water iodine content are takyr soil, yellow soil, grey desert soil, brown desert soil, chernozem and the oasis soil (the most widely distributed Oasis soil in Xinjiang). The soil types with high water iodine content are chestnut, grey-brown desert, gray meadow, and brown calcium soil.

Several limitations need to be addressed. We establish the GWR Model according to the seven influencing factors, determination coefficient *R*^*2*^ of the GWR model is low, the maximum *R*^*2*^ is only 0.38, which is not a satisfactory result. The determination coefficient implies it means that there are some unknown factors not included but possibly important in the model. This study only describes the distribution and aggregation, but does not explore the complete causes of this phenomenon. Further investigation is needed in the future.

## 5 Conclusions

The iodine content level of drinking water in Xinjiang is generally low. Among 94 study areas, 87 areas here have iodine content level that are less than 10 μg/L (92.55%), 68 areas among low water iodine areas have iodine content less than 5 μg/L (72.34%). Only 7 areas have iodine content between 10–40 μg/L (7.45%). Among 3294 sampling points, 58.1% of drinking water iodine content is less than 5 μg/L, 20.29% is between 5–10 μg/L, 18% is between 10–40 μg/L, and 3.4% is more than 40 μg/L. In addition, the distribution of iodine content in water is complex. The distribution usually has a large span in the same area. Furthermore, based on the GWR model, we preliminarily explored the factors affecting the distribution of iodine content in water, and had found that precipitation and soil type are significant influencing factors.
